# Supplementary figures and images for: Quantification of protein abundance and interaction defines a mechanism for operation of the circadian clock
Source: eLife. 2022 Mar 14;11:e73976. doi: 10.7554/eLife.73976 (PMC8983044; doi:10.7554/eLife.73976)

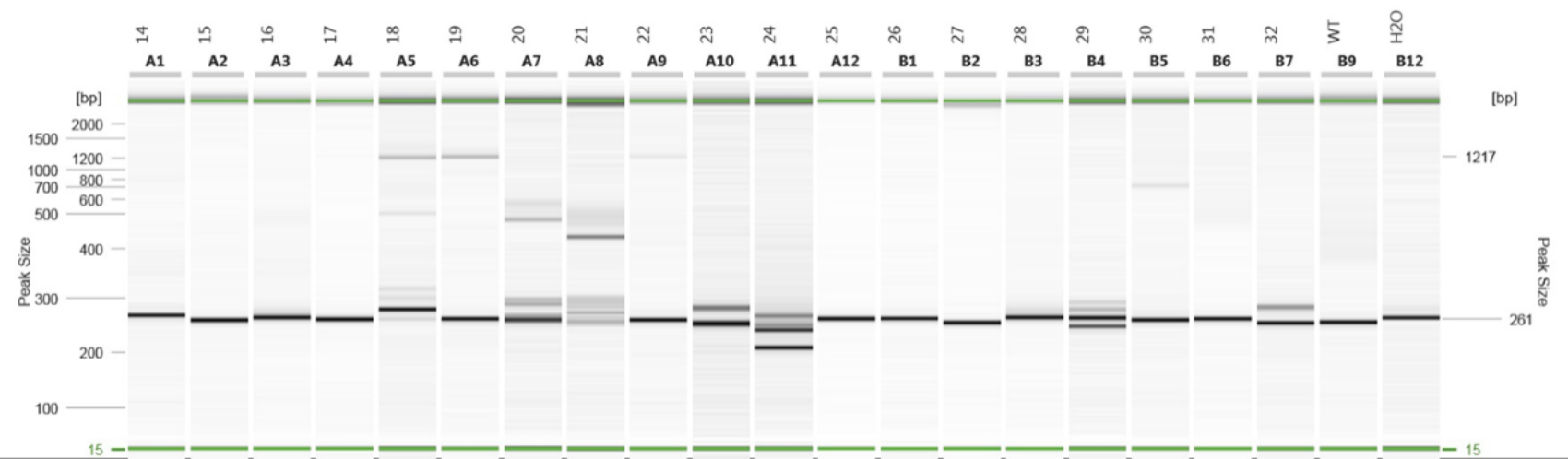

Supplement: Figure 3—figure supplement 1—source data 1. [file elife-73976-fig3-figsupp1-data1.pdf]
